# Supplementary material for: Natural Populations of Astrocaryum aculeatum Meyer in Amazonia: Genetic Diversity and Conservation
Source: Plants (Basel). 2022 Nov 2;11(21):2957. doi: 10.3390/plants11212957 (PMC9655110; doi:10.3390/plants11212957)
Supplement: Supplementary file 1 [file plants-11-02957-s001.zip › Figure S1.pdf]

Natural Populations of *Astrocaryum aculeatum* Meyer in the Amazonia: Genetic Diversity and Conservation

Santiago Linorio Ferreyra Ramos [1], Maria Teresa Gomes Lopes [2], Carlos Meneses [3], Gabriel Dequigiovanni [4], Jeferson Luis Vasconcelos de Macêdo [5], Ricardo Lopes [5], Alexandre Magno Sebbenn [6], Rogério Freire da Silva [3], Therezinha de Jesus Pinto Fraxe [2] and Elizabeth Ann Veasey [7,*]

SUPPORTING INFORMATION

Additional Supporting Information may be found in the online version of this article:

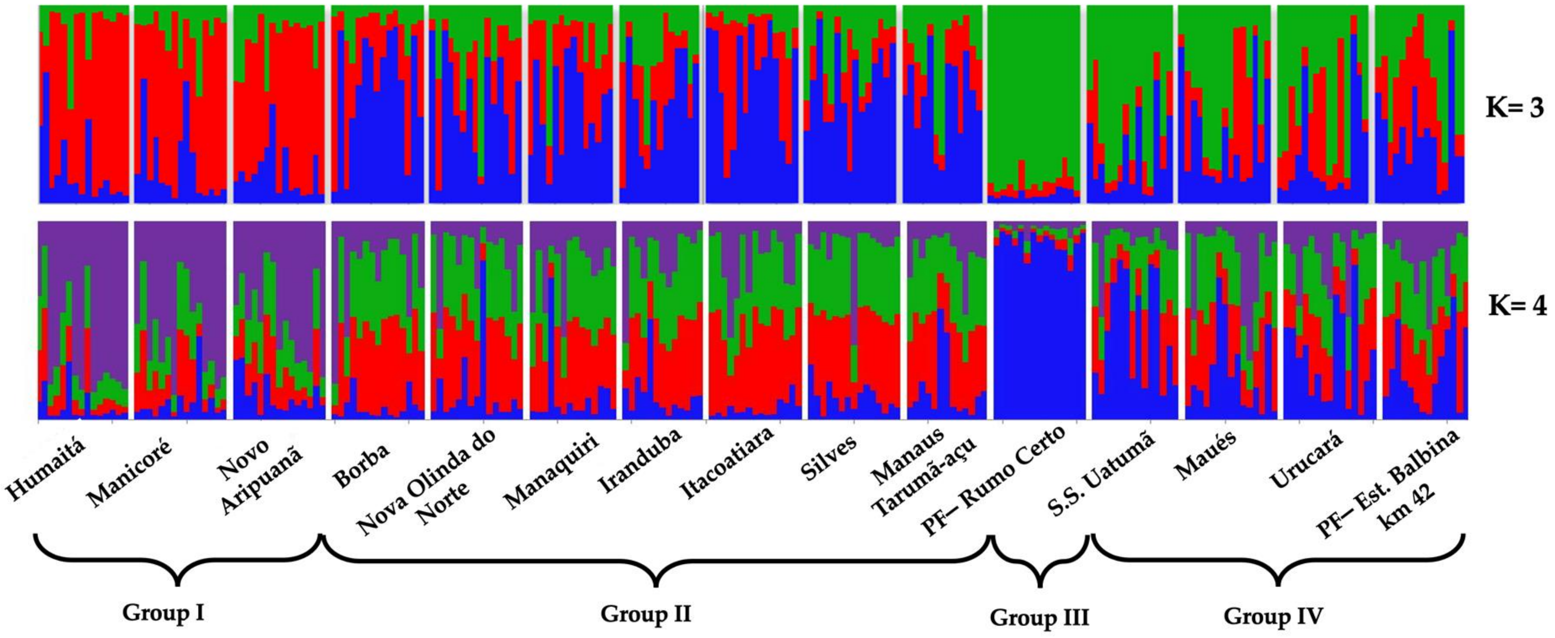

Figure S1 - Analysis of the genetic structure of the 228 matrices selected in the 15 populations of *Astrocaryum aculeatum* from ten microsatellite loci carried out in the program *Structure* indicating that the genotypes were classified into two possible groupings: *K*=3 and *K*=4. The populations were grouped, from left to right, in the direction of the southern, central, and northern regions of Amazonas.
