# Supplementary material for: Natural Populations of Astrocaryum aculeatum Meyer in Amazonia: Genetic Diversity and Conservation
Source: Plants (Basel). 2022 Nov 2;11(21):2957. doi: 10.3390/plants11212957 (PMC9655110; doi:10.3390/plants11212957)
Supplement: Supplementary file 1 [file plants-11-02957-s001.zip › Table S1.pdf]

Natural Populations of *Astrocaryum aculeatum* Meyer in the Amazonia: Genetic Diversity and Conservation

Santiago Linorio Ferreyra Ramos [1], Maria Teresa Gomes Lopes [2], Carlos Meneses [3], Gabriel Dequigiovanni [4], Jeferson Luis Vasconcelos de Macêdo [5], Ricardo Lopes [5], Alexandre Magno Sebbenn [6], Rogério Freire da Silva [3], Therezinha de Jesus Pinto Fraxe [2] and Elizabeth Ann Veasey [7,*]

SUPPORTING INFORMATION

Additional Supporting Information may be found in the online version of this article:

Table S1 Results obtained for the number of combinations with linkage disequilibrium (*CLD*), percentage of *LD* (*LD*%), private alleles, and independently obtained Hardy-Weinberg equilibrium values for each of the ten microsatellite loci in each of 15 populations of *Astrocaryum aculeatum*.

Municipality	C*LD* (*LD*%)	Loci									
		Aac02	Aac03	Aac04	Aac06	Aac07	Aac09	Aac10	Aac11	Aac12	Aac14
Humaitá	-	1.0000	0.0046*	0.1858 [1]	0.0105	0.7748	0.0029* [1]	0.0007*	1.0000	0.0130	0.7137
Manicoré	4 (8.89)	1.0000	0.0138	0.0431	0.2174 [1]	0.1314	0.0012*	0.0009*	1.0000	0.1939	0.7740
Novo Aripuanã	3 (6.67)	1.0000	0.0016*	0.0077	0.7494	0.5401	0.0057 [1]	0.0002*	1.0000	0.0802	0.8715 [1]
Borba	11 (24.44)	1.0000	0.0004*	0.0579	0.9122 [1]	0.0820	0.0000*	0.0002*	1.0000	0.0154	0.3788
Nova Olinda do Norte	5 (11.36)	0.6311 [1]	0.0002*	0.4152	0.0330	0.5504 [1]	0.0003*	0.0044*	1.0000	0.0012*	0.0337
Manaquiri	7 (15.56)	1.0000	0.0005*	0.0355	0.8373	0.7804	0.1262	0.0005*	1.0000	0.0007*	0.9343 [1]
Iranduba	1 (2.22)	1.0000	0.0084	0.4048	0.1389	0.0146	0.0429 [1]	0.0019*	1.0000	0.0065	0.1920
Itacoatiara	6 (13.33)	1.0000	0.0280	0.3060 [1]	0.0203	1.0000	0.0000* [1]	0.5295	1.0000	0.0012*	0.6383

Silves	4 (8.89)	1.0000	0.0003*	0.8120	0.0491	0.8202	0.0174	0.0002*	1.0000	0.0083	0.6473
Maués	4 (8.89)	1.0000	0.0068	0.2930	0.0018*	0.0017*	0.3278	0.0004* [1]	1.0000	0.0246	0.0067
Urucará	3 (6.67)	1.0000 [1]	0.0005*	0.0003*	0.0047*	0.0922	0.0473	0.0003*	1.0000	0.0696	0.8027
S.S. Uatumã	1 (2.22)	1.0000	0.0363	0.0262 [1]	0.0142	0.7855	0.1012	0.0045*	1.0000	0.0011*	0.8288
PF – Rumo Certo	2 (4.44)	1.0000	0.0019*	0.5143	0.8196	0.4096	0.0777 [1]	0.0138	1.0000	0.0008*	0.0435
PF – Est. Balbina km 42	2 (4.44)	1.0000	0.0012*	0.6624	0.7136	0.0630	0.0031*	0.0005*	1.0000	0.0016*	1.0000
Manaus Tarumã-Açú	-	1.0000	0.0057	0.8830	0.3625	0.0432	0.5044	0.0057	1.0000	0.0407	0.0450 [1]

* Deviations from Hardy-Weinberg equilibrium with Fisher's exact test (P<0.005, after fitting for Bonferroni correction (RICE, 1989)); C*LD* = number of combinations with linkage disequilibrium selected with P < 0.0011 after fitting the Bonferroni correction (RICE, 1989); [ ]= number of private alleles.
