# Supplementary material for: Natural Populations of Astrocaryum aculeatum Meyer in Amazonia: Genetic Diversity and Conservation
Source: Plants (Basel). 2022 Nov 2;11(21):2957. doi: 10.3390/plants11212957 (PMC9655110; doi:10.3390/plants11212957)
Supplement: Supplementary file 1 [file plants-11-02957-s001.zip › Table S2.pdf]

Natural Populations of *Astrocaryum aculeatum* Meyer in the Amazonia: Genetic Diversity and Conservation

Santiago Linorio Ferreyra Ramos [1], Maria Teresa Gomes Lopes [2], Carlos Meneses [3], Gabriel Dequigiovanni [4], Jeferson Luis Vasconcelos de Macêdo [5], Ricardo Lopes [5], Alexandre Magno Sebbenn [6], Rogério Freire da Silva [3], Therezinha de Jesus Pinto Fraxe [2] and Elizabeth Ann Veasey [7,*]

SUPPORTING INFORMATION

Additional Supporting Information may be found in the online version of this article:

Table S2. Detailed identification of the collection sites of the 15 populations of *Astrocaryum aculeatum* located in 14 municipalities in the State of Amazonas.

MUNICIPALITY	REFERENCE POINTS	WATERSHEDS	AFFLUENTS OF WATERSHEDS	NUMBER OF MATRICES	GEOGRAPHIC COORDINATES	
					SL	WL
Humaitá	Araçá creek	Madeira river	Araçá creek	1 to 15	–7.80	–62.90
Manicoré	Estrada do Alonso, km 1,5	Madeira river	-	1 to 15	–5.83	–61.27
Novo Aripuana	Estrada NAP 01, km 21	Madeira river	Ariaú river	1 to 15	–5.23	–60.23
Borba	Estrada Borba - Mapia, Km. 18	Madeira river	-	1 to 15	–4.53	–59.58
Nova Olinda do Norte	Estrada vicinal, PA Paquequer, km 12	Madeira river	-	1 to 15	–3.82	–59.02
Manaquiri	Bom Intento Community	Solimões river	-	1 to 14	–3.43	–60.49
Iranduba	Lago do Santo Antonio Community	Solimões river	Santo Antonio lake	1 to 13	–3.26	–60.21
Manaus	Tarumã-açu creek	Negro river	Tarumã-açu creek	1 to 13	–2.89	–60.12

Itacoatiara	Estrada AM 010, km 213	Amazonas river	-	1 to 15	–3.01	–58.81
Urucara	Estrada Urucará-Manari, km 2	Uatumã river	-	1 to 15	–2.51	–57.74
Silves	Livramento Community	Urubu river	Aneba river	1 to 15	–2.94	–58.56
Maués	Estrada Maués Mirim, km 2	Maués-Açu river	-	9 to 15	–3.38	–57.71
Presidente Figueiredo	Estrada da Balbina, km 42	Uatumã river	-	1 to 14	–2.05	–59.67
Presidente Figueiredo	Rumo Certo Community	Uatumã river	Balbina Lake	1 to 15	–1.54	–60.17
São Sebastião do Uatumã	Santa Etelvina (Bacabal) Community	Uatumã river	Maripa river	1 to 14	–2.49	–57.97

SL=South Latitude, WL=West Longitude. SL and WL are shown in decimal degrees.
